# The serodiagnostic potential of recombinant proteins TES–30 and TES–120 in an indirect ELISA in the diagnosis of toxocariasis in cattle, horses, and sheep

**DOI:** 10.1371/journal.pone.0213830

**Published:** 2019-03-14

**Authors:** Lucas Moreira dos Santos, Rafael Amaral Donassolo, Maria Elisabeth Berne, Fábio Pereira Leivas Leite, Luciana Farias da Costa Avila, Carlos James Scaini, Ângela Nunes Moreira, Fabricio Rochedo Conceição

**Affiliations:** 1 Department of Biotechnology, Universidade Federal de Pelotas, Pelotas, RS, Brasil; 2 Department of Parasitology, Universidade Federal de Pelotas, Pelotas, RS, Brasil; 3 Faculty of Medicine, Universidade Federal do Rio Grande, Rio Grande, RS, Brasil; Academic Medical Centre, NETHERLANDS

## Abstract

Toxocariasis is a zoonotic disease that affects humans and animals alike. Although recombinant proteins are widely used for its diagnosis in humans, their performance in companion and production animals remains unknown. This study aimed to investigate the serodiagnostic potential of the recombinant proteins rTES–30 and rTES–120 from *Toxocara canis* in an indirect ELISA for cattle, horses, and sheep. Serum samples collected from the animals were tested with indirect ELISA and Western Blotting using *T*. *canis* TES–30 and TES–120 recombinant proteins produced in *Escherichia coli*, as well as native-TES. In the ELISA, rTES–30 showed high serodiagnostic potential in sheep and horses (92.6% and 85.2%, respectively), while the sensitivity of rTES–120 was higher in cattle and horses (97.2% and 92.6%, respectively). Furthermore, a highly positive association was observed between native and recombinant proteins in seropositive samples, while a moderately positive association was observed in seronegative samples, probably due to the lower specificity of native TES. In conclusion, our study indicates that the use of recombinant proteins in an indirect ELISA is an effective tool for the serodiagnosis of toxocariasis in animals, with the choice of protein being species-dependent.

## Introduction

Endoparasites are among the important threats to the health of companion and production animals, whereby infections diminish the economic value of the animals [1]. Among infections caused by endoparasites, toxocariasis is important. It is transmitted through the infectious eggs of *Toxocara* sp., and its symptomatology varies depending on the larval migration and parasite and host species. For example, *Toxocara vitulorum* infection in ruminants was detected in 21.1% out of 819 animals in Qinghai Tibetan Plateau, China, and was associated with increased morbidity and mortality, causing important economic loss to the farmers [2,3].

Companion and production animals such as horses and sheep, that commonly share ambient surroundings with definitive hosts (canids and cattle), are prone to become paratenic hosts of the parasite, acting as vectors in the spread of the parasite to a vast number of species and adversely affecting the economy of the region [4,5].

The clinical diagnosis of toxocariasis is difficult as most manifestations are non-specific [6]. Therefore, the only viable diagnostic options are laboratory-based and depend on whether the host is definitive or paratenic, whereby fecal examination and enzyme-linked immunosorbent assay (ELISA) in combination with western blotting (WB) are used [2,6]. The fecal examination is time-consuming and requires expertise to produce a viable diagnosis. Furthermore, depending on the degree of infection, it has low sensitivity, producing more false negatives than serological methods [7,8].

In contrast, ELISA and WB are indirect assays, but are rapid, accessible, and require minimum training [9]. However, the *Toxocara* excretion-secretion protein family (TES), obtained from the larval culture, is required for these assays, which is a laborious process and also presents a number of cross-species reactions, especially with other helminths commonly found in livestock, resulting in an erroneous diagnosis [10–12]. In order to find an alternative, several studies have investigated the potential of recombinant proteins for the diagnosis of toxocariasis in an attempt to reduce the time, cost, and cross-reactivity against native TES [13–16].

This study investigates the serodiagnostic potential of *T*. *canis* TES–30 and TES–120 recombinant proteins in an indirect ELISA for the detection of toxocariasis in animals. This technique could represent an important breakthrough in increasing the specificity of serodiagnosis and facilitating a rapid and precise diagnosis.

## Materials and methods

### Cloning and expression of recombinant proteins

The recombinant proteins were cloned, expressed and purified as described in our previous studies [17–18].

### Production of native TES

Adult worms of *T*. *canis* were obtained by treatment of young (4–8 weeks-old) dogs with 15 mg/kg pyrantel pamoate. The female parasites were subjected to hysterectomy to obtain parasite eggs, which were incubated for 28 days in 2% formalin at 28°C to allow the formation of embryos [19].

The native TES antigens were prepared as described by Savigny (1979) [20]. In summary, the larvae of *T*. *canis* were grown in RPMI 1640, and the medium was collected every three days, pooled, and centrifuged. The supernatant was filtered through a 0.2 μm filter (Sigma Aldrich, USA) into a dialysis tube (molecular weight cut-off of 6.000–8.000 Da; Sigma Aldrich, USA). The solution was dialyzed against 250 volumes of distilled water at 4°C. After dialysis, the supernatant was concentrated in a vacuum concentrator, reconstituted in distilled water, and stored in aliquots at −70°C. The proteins were further quantified using a Pierce BCA kit (Thermo Fisher Scientific, USA).

### Sample collection

The samples were collected from three sera panels from all randomly selected male and female animals aged more than one year. Initial (prior to this study) blood collection was performed from the jugular vein of animals, using a vacutainer tube. The serum samples were stored at –20°C until use. Sera panel-1 consisted out of 104 non-immunized sheep samples that were randomized from a total of 1,642 samples collected from 95 farms across 21 countries [5]. Sera panel-2 consisted out of samples from 46 non-immunized *Bos taurus* cattle that were collected for the study by Cunha *et al*. (2012) [21]. Sera panel-3 consisted out of samples from 38 non-immunized horses used for the study by Moraes *et al*. (2014) [22].

For the negative controls for serodiagnosis of each animal species, negative sera samples were selected from the sera banks of each species that were maintained by the UFPel Parasitology Laboratory. These samples were collected from animals that tested negative in the excretory–secretory *Toxocara canis* (TES) antigen enzyme-linked immunosorbent assay (ELISA) and were born in farms where there were no dogs. Fetal sera were also used as negative controls. Positive sera (positive controls) were collected from two adult animals of each species following experimental vaccination with rTES–30 and rTES–120 (400 ng) by the subcutaneous application.

### Western blotting (WB) assay

The WB assay was used in two phases during the study. First, it was used to test the samples in order to distinguish negative sera from positive sera. Secondly, it was used to confirm ELISA results close to the cut-off absorbance (borderline).

The following method was followed in both the WB assays: 20 μg/mL each of rTES–30 and rTES–120 were electrophoresed on a 12% SDS-PAGE and electrotransferred onto a nitrocellulose membrane (GE Healthcare Life Sciences, USA) using a transblot apparatus (Bio-Rad, USA) overnight at 4°C. The transfer of proteins to the membrane was confirmed using Ponceau S staining (Sigma-Aldrich, USA). The membrane was cut into strips and blocked using 5% dry-milk (Nestle, Sweden) in PBS-T for 1 h. The strips were then incubated with sera samples (diluted 1:200 in PBS-T) overnight at 4°C, followed by incubation with anti-horse IgG (whole molecule) horseradish peroxidase (Sigma Aldrich, USA), anti-sheep IgG (whole molecule) horseradish peroxidase (Sigma Aldrich, USA), or anti-bovine IgG (whole molecule) horseradish peroxidase (Sigma Aldrich, USA) at an optimized dilution (1:10000, 1:5000, or 1:2500, respectively) in PBS-T, and incubated for 3 h at room temperature. The strips were washed with PBS-T for 5 min each between each step. Finally, DAB Solution (0.025% 3,3′-diaminobenzidine, 0.0009% H_2_O_2_, and 0.05 M Tris/HCl-solution, Sigma Aldrich, USA) was used to develop the blots.

### Indirect ELISA

ELISA was used to verify the antigenicity of the proteins. The ELISA protocol was optimized prior to the study. Each well of the 96-well flat-bottomed microtiter plate (Nunc Immuno Maxisorp, Thermo Fisher Scientific, USA) was coated with 100 μL of each antigen at the optimum concentration (50 ng) in 0.02 M bicarbonate buffer, pH 9.6. The plates were then covered and incubated overnight at 4°C. The plates were washed with PBS-T to remove unattached antigens. The plates were washed thrice for 5 min each time with PBS-T, and then each well was blocked with 5% dry-milk (Nestle, Sweden) in PBS-T solution for 1 h at 37°C. The plates were again washed as previously described, followed by the addition of sera samples (100 μL, 1:150 in PBS-T, duplicate wells) and incubation for 1 h at 37°C. After the washing step, anti-horse IgG (whole molecule) horseradish peroxidase (Sigma Aldrich, USA), anti-sheep IgG (whole molecule) horseradish peroxidase (Sigma Aldrich, USA), or anti-bovine IgG (whole molecule) horseradish peroxidase (Sigma Aldrich, USA) were added at optimized dilutions (1:10000, 1:5000, 1:2500, respectively) in PBS-T, and incubated for 1 h at 37°C. After a final washing step, o-phenylenediamine dihydrochloride substrate (Sigma Aldrich, USA) was added, and after 15 min, the ODs were measured at 450 nm using an ELISA spectrophotometer (Biochrom EZ Read 400, United Kingdom). The OD readings were adjusted with PBS-T as blank, and the cut-off value was used to distinguish between the positive and negative results. These cut-off values were based on the results of Receiver Operating Characteristic (ROC) statistical analysis (S1–S3 Files). The cut-off values were: cattle- 0.332 (rTES–30) and 0.414 (rTES–120); horses- 0.099 (rTES–30) and 0.189 (rTES–120); and sheep- 0.499 (rTES–30) and 0.4015 (rTES–120).

### Statistical analysis

The data were statistically analyzed using Pearson's chi-square and Pearson's correlation matrix (for qualitative variables, seropositive and seronegative); two-way ANOVA (for quantitative variables, OD readings); and ROC curve (to determine cut-off values) using the statistical software GraphPad Prism version 7.

The cut-off values were calculated using the method described by Hanley (1982) [23], by plotting sensitivity against specificity. The negative and positive samples were added on the software based on the results from the initial WB. The cut-off for each animal species was chosen based on the likelihood ratio using the method described by Johnson (2004) [24].

### Ethics

This retrospective study was previously approved by the Federal University of Pelotas Ethical Research Committee under protocol CEEA 2133 [5,21,22].

## Results and discussion

Yield, size, and purification of the recombinant proteins were as described in our previous studies [17–18]. Similar to Farmer *et al*. (2017) study, these proteins were stored in urea buffer [25].

The sensitivity and specificity of the recombinant proteins in the indirect ELISA are presented in Tables 1–3. There was no benefit associated with the use of both proteins at the same time (*p* = 0.9313), in any of the animal serodiagnostic assays studied.

**Table 1:** ELISA sensitivity and specificity for each recombinant protein as determined by a Receiver Operating Characteristic (ROC) analysis for cattle.

Parameter	rTES-30	rTES-120
Positive samples (sensitivity)	14/36 (38.89%)	35/36 (97.22%)
Sensitivity 95% confidence interval	23.14% - 56.54%	85.47% - 99.93%
Negative samples (specificity)	9/10 (90.00%)	9/10 (90.00%)
Specificity 95% confidence interval	55.5% - 99.75%	55.5% - 99.75%
Cut-off	> 0.332	> 0.414
*p* value	< 0.001	

**Table 2:** ELISA sensitivity and specificity for each recombinant protein as determined by a Receiver Operating Characteristic (ROC) analysis for horses.

Parameter	rTES-30	rTES-120
Positive samples (sensitivity)	23/27 (85.19%)	25/27 (92.59%)
Sensitivity 95% confidence interval	66.27% - 95.81%	75.71% - 99.09%
Negative samples (specificity)	10/11 (90.91%)	10/11 (90.91%)
Specificity 95% confidence interval	58.72% - 99.77%	58.72% - 99.77%
Cut-off	> 0.099	> 0.189
*p* value	0.6687	

**Table 3:** ELISA sensitivity and specificity for each recombinant protein as determined by a Receiver Operating Characteristic (ROC) analysis for sheep.

Parameter	rTES-30	rTES-120
Positive samples (sensitivity)	25/27 (92.59%)	15/27 (55.56%)
Sensitivity 95% confidence interval	79.7% - 96.92%	35.33% - 74.52%
Negative samples (specificity)	73/77 (94.81%)	76/77 (98.7%)
Specificity 95% confidence interval	90.02% - 97.73%	92.98% - 99.97%
Cut-off	> 0.499	> 0.401
*p* value	0.0041	

**Fig 1:**
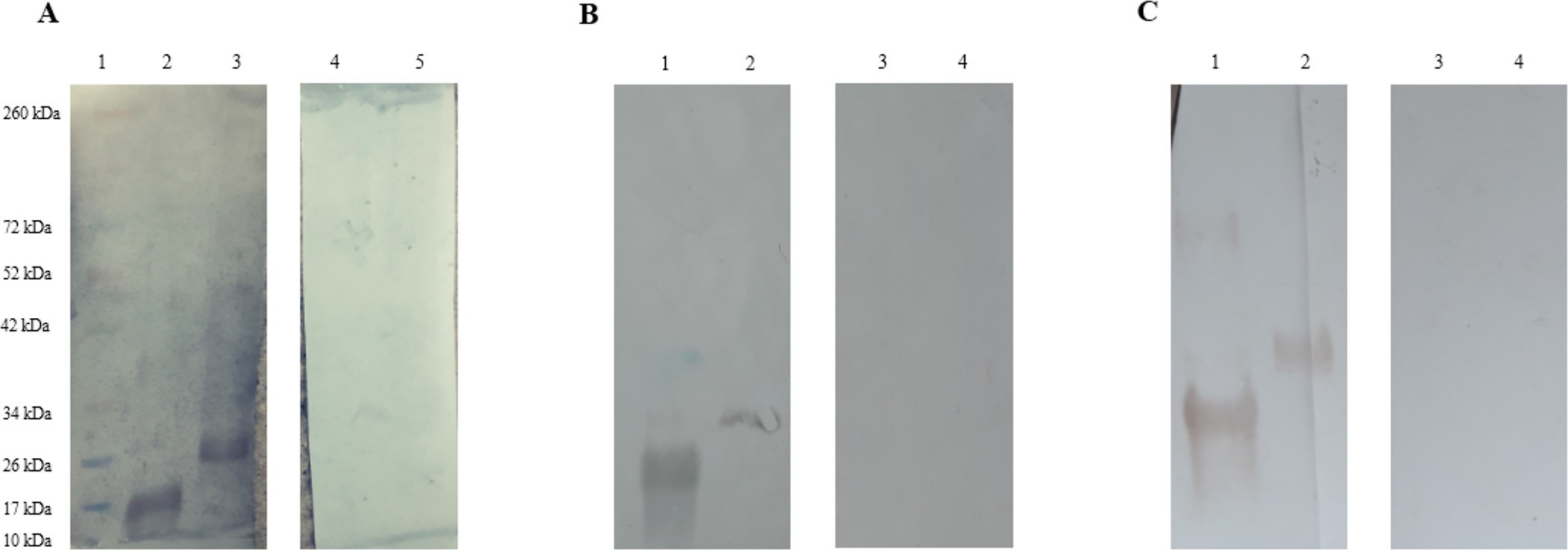
**(A) Bovine ELISA close to cut-off confirmation via WB assay.** Lane 1: PageRuler™ Pre-stained Protein Ladder (Thermo Fisher Scientific, USA); Lane 2: rTES–30 with pooled sera from four seropositive animals; Lane 3: rTES–120 with pooled sera from four seropositive animals; Lane 4: rTES–30 with pooled sera from four seronegative animals; Lane 5: rTES–120 with pooled sera from four seronegative animals. **(B) Horse ELISA close to cut-off, confirmed by the WB assay.** Lane 1: rTES–30 with pooled sera from four seropositive animals; Lane 2: rTES–120 with pooled sera from four seropositive animals; Lane 3: rTES–30 with pooled sera from four seronegative animals; Lane 4: rTES–120 with sera from four seronegative animals; **(C) Sheep ELISA close to cut-off, confirmed by the WB assay.** Lane 1: rTES–30 with pooled sera from four seropositive animals; Lane 2: rTES–120 with pooled sera from four seropositive animals; Lane 3: rTES–30 with pooled sera from four seronegative animals; Lane 4: rTES–120 with pooled sera from four seronegative animals.

The confirmation of the ELISA cut-off can be seen in Fig 1. Despite the lower sensitivity of the ELISA in case of some recombinant proteins and animal combinations, the WB assay permitted the visualization of both bands in the seropositive sera.

In this study, we assessed the performance of a proposed alternative (recombinant TES in an indirect ELISA) method for the serodiagnosis of toxocariasis. Our methodology for the serodiagnosis of toxocariasis was based on our previous study [18] and that of Nguyen *et al*. [26,27] study. In both the studies, TES–30 (in recombinant and native forms) was demonstrated to be the most specific biomarker for the serodiagnosis of toxocariasis in paratenic animals and humans [18,27]. Although we had evaluated different species in the earlier study and this study has employed recombinant proteins for the serodiagnosis of toxocariasis in production animals for the first time, we have applied the best available tool for the serodiagnosis of unknown samples, in addition to positive and negative controls.

One of the major problems with the diagnosis of toxocariasis using native TES is the lack of specificity, because of the presence of other families of helminths that are common parasites in production animals [10,11,28]. In humans, the diagnosis of toxocariasis not only requires a diagnostic laboratory but also epidemiological and clinical data to avoid misdiagnosis, which are unavailable in production animals [9]. To overcome this obstacle, we employed an extra method to avoid false positives and false negatives, wherein we assayed each sample with recombinant proteins in a blotting assay instead of using ELISA as the starting point. This extra step permitted the confirmation of seronegatives, making the ELISA cut-off viable. Finally, only samples that were above the cut-off with one recombinant protein, as well as native TES, were considered seropositive, thus nullifying the possible low sensitivity of recombinant proteins and the low specificity of native TES altogether.

Overall, we observed a high specificity with the recombinant proteins, concordant with the study of Mohamad *et al*. [13]. In addition, these proteins (rTES–30 and rTES–120) had been previously tested against ascariasis, trichuriasis, ancylostomids, strongyloidiasis, hymenolepiasis, and fasciolosis in humans without any cross-reactions [17]. However, the production animals are in close contact with the environment and human specificity should not be extrapolated to animal specificity, as different parasites could introduce new specificity issues. The issue of specificity is yet not to be completely explored and should be studied further [29,30,31].

It is worth noting that we chose not to adsorb the sera with an *Ascaris suum* antigen in an attempt to achieve a better specificity in native TES [11]. We made the decision to compare the absolute efficacy of recombinant against native TES because adding another antigen to the procedure in the disease diagnosis of production animals would have increased the diagnosis-related cost further, which is an important factor for farmers [31]. Moreover, the adsorption of *Ascaris suum* antigens only prevents cross-reactions with the *Ascarididae* family; hence, cross-reactivity issues with fascioliasis and strongyloidiasis infections would remain [32,33].

ELISA sensitivities with recombinant proteins in different animal species were highly variable. In horses, ELISA with recombinant proteins had a high sensitivity, while rTES–30 ELISA was more sensitive in sheep and rTES–120 ELISA was better for bovine serodiagnosis. As the recombinant proteins are significantly different in amino acids structure, we suggest that the immune systems of different species react differently to each protein, perhaps due to the differences in pathogeny and proteomic profile that the *Toxocara* larvae present in the physiological and immunological functions of each animal species [34]. For example, our previous study reported that rTES–30 appeared to be the only viable tool for serodiagnosis in mice [18].

rTES–30 has been widely used as a specific biomarker for toxocariasis in paratenic hosts. This is in agreement with our results, where rTES–30 showed a better sensitivity in both sheep and horse [25,27]. rTES-30 ELISA has a high sensitivity towards *T*. *canis* and *T*. *cati* infections in the paratenic hosts, being capable of the serodiagnosis of both parasites, although sensitivity against *T*. *vitulorum* and *T*. *malaysiensis* remains unknown [35,36].

On the other hand, rTES–120 performed better with cattle, which could be influenced by the fact that cattle are definitive hosts of *T*. *vitulorum*. Therefore, rTES–120 could potentially be related to the full development of the larvae; however, more studies are required to support this conclusion [14,35,36].

In this study, we could not confirm the actual disease, because we did not conduct biopsies on the paratenic hosts or fecal examinations on the definitive hosts. As toxocariasis is a chronic disease with active and dormant larvae phases, infected animals could be misdiagnosed as false negatives in biopsies or fecal examination which are assays that have lower sensitivity in this disease [8]. In these cases, WB with TES–30 has been shown to be the most sensitive and specific method for this purpose [27]. Nevertheless, the main objective of this study was to assess a potentially rapid, feasible, and inexpensive tool for the serodiagnosis of toxocariasis for an entire farm animal population, for which we compared the efficacy of our methods against the standard method (ELISA TES).

## Conclusions

In conclusion, our study indicates that it is beneficial to use recombinant proteins as a substitute for the laborious native-TES. However, selection of the recombinant protein to be used may depend on the animal species being diagnosed.

## Supporting information

S1 File ELISA and cut-off values for native and recombinant TES in cattle. File containing the enzyme-linked immunosorbent assay absorbances, the receiver operating characteristic analysis and the resulting cut-offs for toxocariasis diagnosis using native and recombinant *Toxocara* excretion-secretion proteins in cattle.
(XLSX)

S2 File ELISA and cut-off values for native and recombinant TES in sheep. File containing the enzyme-linked immunosorbent assay absorbances, the receiver operating characteristic analysis and the resulting cut-offs for toxocariasis diagnosis using native and recombinant *Toxocara* excretion-secretion proteins in sheep.
(XLSX)

S3 File ELISA and cut-off values for native and recombinant TES in horses. File containing the enzyme-linked immunosorbent assay absorbances, the receiver operating characteristic analysis and the resulting cut-offs for toxocariasis diagnosis using native and recombinant *Toxocara* excretion-secretion proteins in horses.
(XLSX)
